# HeteroMeth: A Database of Cell-to-cell Heterogeneity in DNA Methylation

**DOI:** 10.1016/j.gpb.2018.07.002

**Published:** 2018-09-06

**Authors:** Qing Huan, Yuliang Zhang, Shaohuan Wu, Wenfeng Qian

**Affiliations:** 1 State Key Laboratory of Plant Genomics, Institute of Genetics and Developmental Biology, Chinese Academy of Sciences, Beijing 100101, China; 2 Key Laboratory of Genetic Network Biology, Institute of Genetics and Developmental Biology, Chinese Academy of Sciences, Beijing 100101, China; 3 University of Chinese Academy of Sciences, Beijing 100049, China

**Keywords:** Cell-to-cell heterogeneity, DNA methylation, Bisulfite sequencing, Single cell, Shannon entropy

## Abstract

**DNA methylation** is an important epigenetic mark that plays a vital role in gene expression and cell differentiation. The average DNA methylation level among a group of cells has been extensively documented. However, the **cell-to-cell heterogeneity** in DNA methylation, which reflects the differentiation of epigenetic status among cells, remains less investigated. Here we established a gold standard of the cell-to-cell heterogeneity in DNA methylation based on single-cell **bisulfite sequencing** (BS-seq) data. With that, we optimized a computational pipeline for estimating the heterogeneity in DNA methylation from bulk BS-seq data. We further built HeteroMeth, a database for searching, browsing, visualizing, and downloading the data for heterogeneity in DNA methylation for a total of 141 samples in humans, mice, Arabidopsis, and rice. Three genes are used as examples to illustrate the power of HeteroMeth in the identification of unique features in DNA methylation. The optimization of the computational strategy and the construction of the database in this study complement the recent experimental attempts on single-cell DNA methylomes and will facilitate the understanding of epigenetic mechanisms underlying cell differentiation and embryonic development. HeteroMeth is publicly available at http://qianlab.genetics.ac.cn/HeteroMeth.

## Introduction

DNA methylation is a heritable epigenetic mark that has a strong impact on gene expression and plays a vital role in genomic imprinting, cell differentiation, X chromosome inactivation, and transposon silencing [1], [2], [3], [4], [5]. The average intensity of methylation in a DNA region among a group of cells (DNA methylation level) can be quantified by whole-genome bisulfate sequencing (BS-seq), in which sodium bisulfate converts cytosine to uracil (and subsequently to thymine during PCR) but leaves methylated cytosine unaffected. Therefore, DNA methylation level can be estimated by the fraction of converted cytosine in the bisulfate-treated DNA samples [6].

Although DNA methylation level has been extensively studied [1], [2], [3], [4], [5], [7], [8], [9], the heterogeneity in DNA methylation among individual cells remains less investigated [10]. It has been increasingly recognized that the cell-to-cell heterogeneity in DNA methylation plays an important role in cell differentiation and embryonic development because it establishes transcriptomic variation among isogenic cells [11], [12], [13], [14], [15]. In addition, the cell-to-cell heterogeneity in DNA methylation may also play a role in generating heterogeneity among tumor cells [16].

Both experimental and computational strategies have been developed to quantify the cell-to-cell heterogeneity in DNA methylation. For example, BS-seq has been adapted to individual cells. However, single-cell BS-seq (scBS-seq) remains technically challenging and cost-intensive, and therefore it had only been applied to a small number of studies [11], [12], [13], [14], [15]. Computational strategies based on bulk BS-seq data have also been developed. Since the methylation status (methylated or unmethylated) of several consecutive CpG sites can be accessed in a single BS-seq read, the complexity of the DNA methylation pattern in a cell population can be determined from individual sequencing reads in the bulk BS-seq experiment [17]. However, such computational strategies may suffer from an overestimation of the cell-to-cell heterogeneity, because technical errors in the BS-seq experiments (*e.g.*, the conversion efficiency of sodium bisulfite, PCR errors and biases, and sequencing errors) cannot be distinguished from the genuine heterogeneity among cells. The extent of such overestimation has not been examined in experiments.

In this study, we established a gold standard of the cell-to-cell heterogeneity in DNA methylation from scBS-seq data, based on which we fine-tuned the computational approaches. With that, we further processed a large number of bulk BS-seq datasets from humans, mice, Arabidopsis, and rice. Finally, we built a database HeteroMeth (standing for heterogeneity in DNA methylation), where the data for heterogeneity in DNA methylation from 4 species can be retrieved and compared. This database will significantly contribute to the understanding of the epigenetic mechanisms underlying the differentiation of cells and the development of organisms.

## Results and discussion

### Establishing a gold standard of the heterogeneity in DNA methylation from scBS-seq data

**Figure 1:**
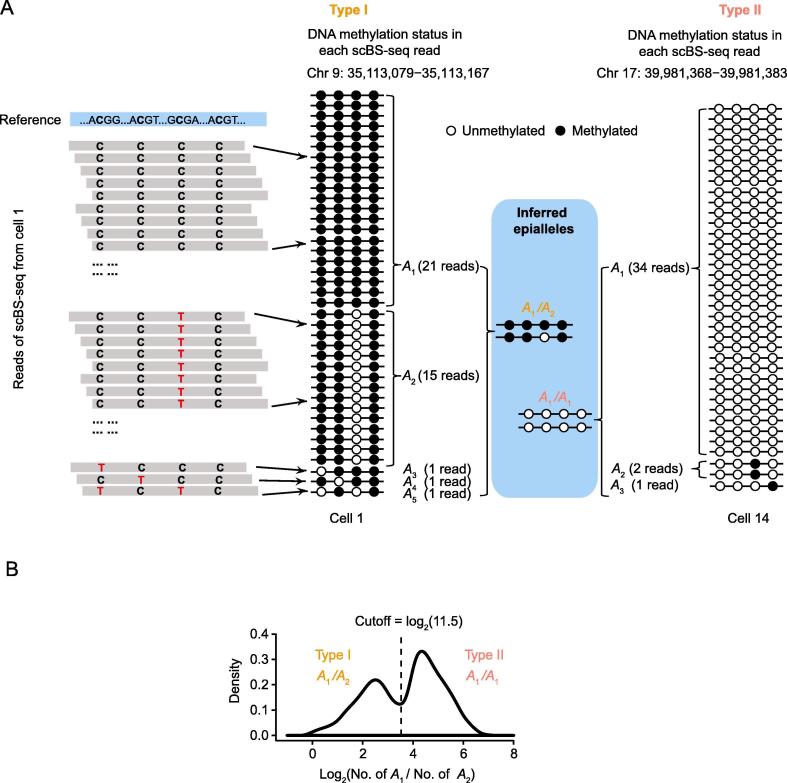
**A gold standard of the cell-to-cell heterogeneity in DNA methylation** **A.** Representative scBS-seq reads from cell 1 that were mapped to the mouse genome (Chr 9: 35,113,079–35,113,167) are shaded. Cytosine in a BS-seq read represents a methylated CpG site, whereas thymine represents an unmethylated CpG site. Examples of heterozygous (*A*_1_/*A*_2_, Chr 9: 35,113,079–35,113,167 in cell 1) and homozygous (*A*_1_/*A*_1_, Chr 17: 39,981,368–39,981,383 in cell 14) epigenetic status are shown, respectively. The identity and number of all the methylation patterns in a cell are shown. Circles represent 4 consecutive CpG sites in a DNA segment, among which closed ones represent methylated sites and open ones represent unmethylated sites. Methylation patterns are ranked by their frequencies and *A_i_* represents the *i*th methylation pattern. **B.** The distribution of the log_2_-transformed frequency ratio between the top 2 methylation patterns. The dashed line indicates the cutoff of the frequency ratio. If there is only one methylation pattern present in a DNA segment, a “pseudo” methylation pattern was added with a read count equal to 1.

To develop a computational method that can gauge the cell-to-cell heterogeneity in DNA methylation from bulk BS-seq data, we attempted to establish a gold standard based on scBS-seq data. To this end, we retrieved the scBS-seq reads from 20 mouse embryonic stem cells (mESCs) [14]. All reads uniquely mapped to a DNA segment that covers at least 4 methylation sites were used for subsequent analyses. To determine the identities of the two epialleles in a diploid cell, we identified the top 2 methylation patterns (*A*_1_ and *A*_2_, respectively) of a DNA segment in each cell based on their frequencies among all reads mapped to this DNA segment (Figure 1A). The log_2_-transformed frequency ratio between the top 2 methylation patterns exhibited a bimodal distribution (Figure 1B), in which the left peak indicated a heterozygous state of two epialleles (*A*_1_/*A*_2_) and the right one indicated a homozygous state (*A*_1_/*A*_1_). In the latter scenario, *A*_2_ was observed with low frequency in the scBS-seq data, likely due to technical errors. The cutoff of the frequency ratio was set to 11.5 (Figure 1B), based on which 57% of DNA segments are epigenetically heterozygous in a cell (Figure 1B).

**Figure 2:**
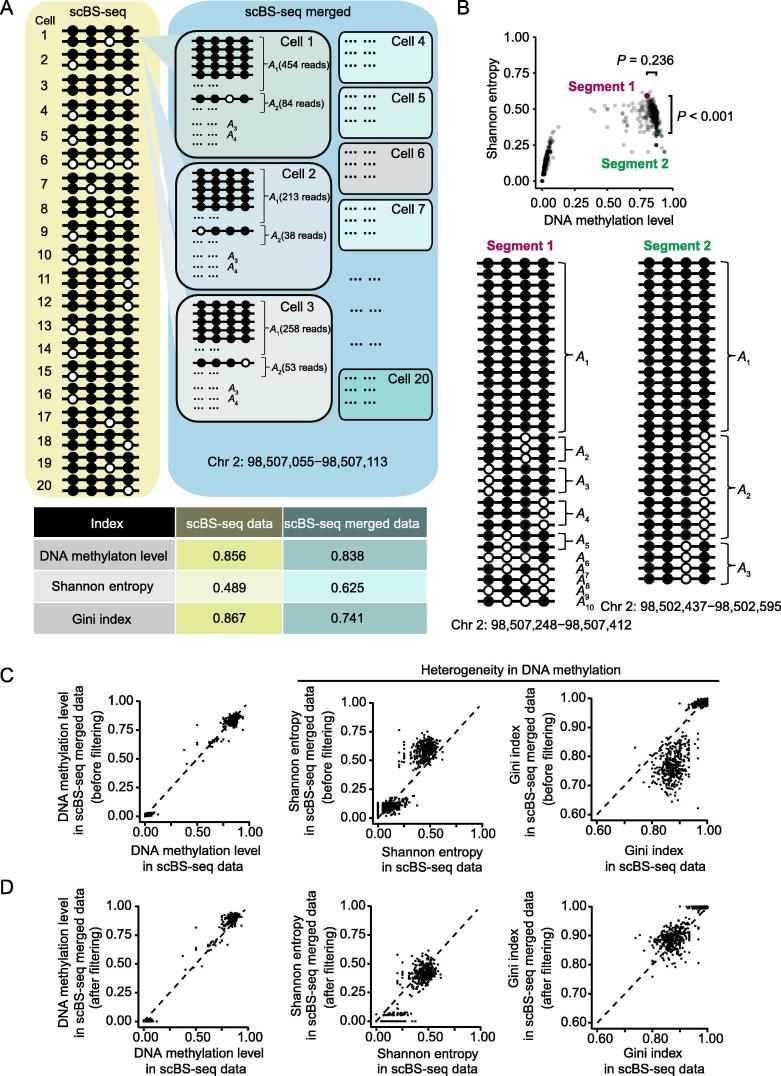
**Reproducing the gold standard from the *in silico* merged scBS-seq data** **A.** Heterogeneity in DNA methylation estimated from the 40 epialleles that were identified in the mouse scBS-seq data of a DNA segment (Chr 2: 98,507,055–98,507,113). DNA methylation level and heterogeneity (Shannon entropy and Gini index) of this segment are provided for both scBS-seq data (40 epialleles identified from 20 cells) and the *in silico* merged scBS-seq data (all sequencing reads from 20 cells), respectively. **B.** An example of two DNA segments (Chr 2: 98,507,248–98,507,412 and Chr 2: 98,502,437–98,507,595) that exhibit similar DNA methylation levels but exhibit different extents of heterogeneity. Each dot represents a DNA segment that contains 4 consecutive CpG sites. The epialleles identified in single cells are provided (the purple and green dots). Note that the DNA methylation status was not identified in every single cell due to the limited sequencing depth in scBS-seq. *P* values were calculated from the permutation test. **C.** Heterogeneity calculated from the unfiltered merged data. The dashed line represents *y* = *x*. Heterogeneity calculated from the merged data is overestimated (greater Shannon entropies and smaller Gini indices). **D.** The gold standard can be faithfully reproduced from the merged data with the filtered data.

For the DNA segment shown in Figure 2A, we calculated two parameters that reflect heterogeneity in DNA methylation, Shannon entropy and Gini index, from 40 epialleles identified from 20 cells, and used them as the gold standard of the heterogeneity in DNA methylation. Segments exhibiting similar DNA methylation levels may exhibit various extents of heterogeneity in DNA methylation (Figure 2B). For example, two segments shown in Figure 2B exhibited a significant difference in Shannon entropy (*P* < 0.001, permutation test, Figure S1).

Note that the heterogeneity calculated here comprises both the cell-to-cell heterogeneity and the epiallelic heterogeneity within a cell. To determine whether the total heterogeneity in DNA methylation can be used as a proxy for the cell-to-cell heterogeneity, we concatenated the two epialleles of 4 consecutive DNA methylation sites in a cell (*i.e.*, 8 methylation sites) and calculated Shannon entropy from them. The total heterogeneity (calculated based on 4 consecutive methylation sites of 40 epialleles) and the cell-to-cell heterogeneity (calculated based on 8 “consecutive” methylation sites of 20 cells) are highly correlated (*r* = 0.96, *P* < 10^−16^, Pearson’s correlation), suggesting that the total heterogeneity is a good predictor of the cell-to-cell heterogeneity.

### Reproducing the gold standard from *in silico* merged scBS-seq data and from bulk BS-seq data

To examine whether the gold standard can be reproduced from bulk BS-seq data, we first *in silico* merged all sequencing reads from the scBS-seq data of these 20 mESCs (single-cell merged) and calculated Shannon entropy and Gini index from these reads (Figure 2A). Not unexpectedly, the heterogeneity calculated was higher in the merged data (Figure 2C), because the methylation patterns that were discarded in the scBS-seq gold standard (*A*_3_, *A*_4_… in Figure 1A and Figure 2A) were used in the calculation of the merged data. To eliminate this effect, we removed the low-frequency methylation patterns in the merged data that likely reflect technique errors (*e.g.*, incomplete bisulfite conversion, PCR errors, and sequencing errors), and found that with a frequency cutoff of 1/32, the heterogeneity in the gold standard can be faithfully reproduced from the *in silico* merged data (Figure 2D).

**Figure 3:**
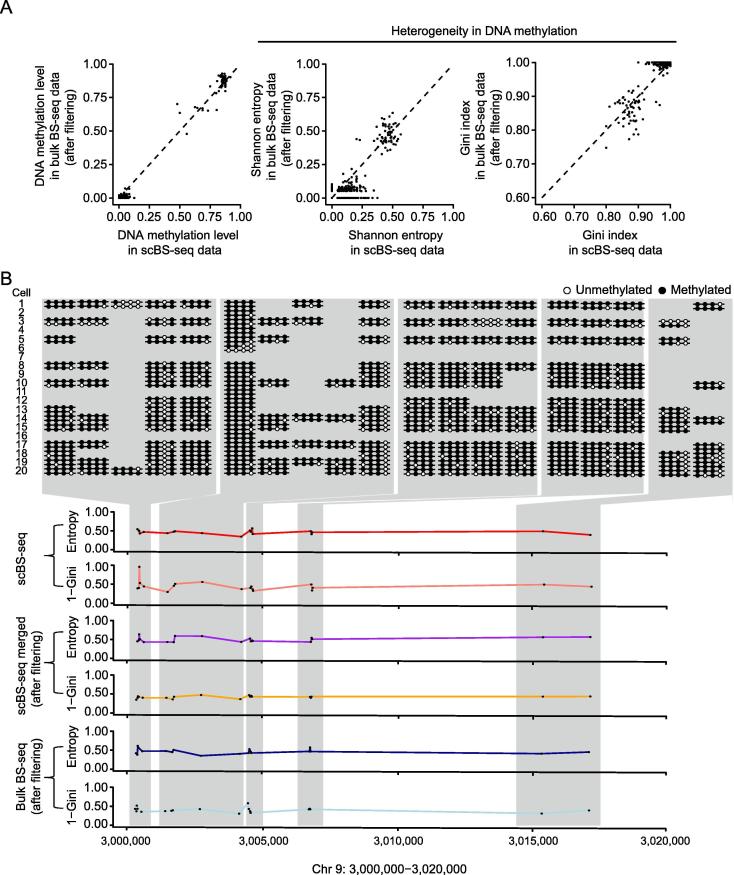
**Reproducing the gold standard from the bulk BS-seq data** **A.** The gold standard can be faithfully reproduced from the corresponding filtered bulk BS-seq data. **B.** The landscape of heterogeneity in DNA methylation (Chr 9: 3,000,000–3,020,000) is largely reproduced from the filtered *in silico* merged scBS-seq and the filtered bulk BS-seq data.

Bulk BS-seq experiment was also performed for the same batch of mESCs. With the same frequency cutoff of methylation patterns (1/32), the heterogeneity of DNA methylation can be accurately estimated from the bulk BS-seq data (Figure 3A). The landscape of heterogeneity in DNA methylation in a region of chromosome 9 is shown as an example (Figure 3B).

### HeteroMeth: A database of cell-to-cell heterogeneity in DNA methylation calculated from bulk BS-seq data

**Figure 4:**
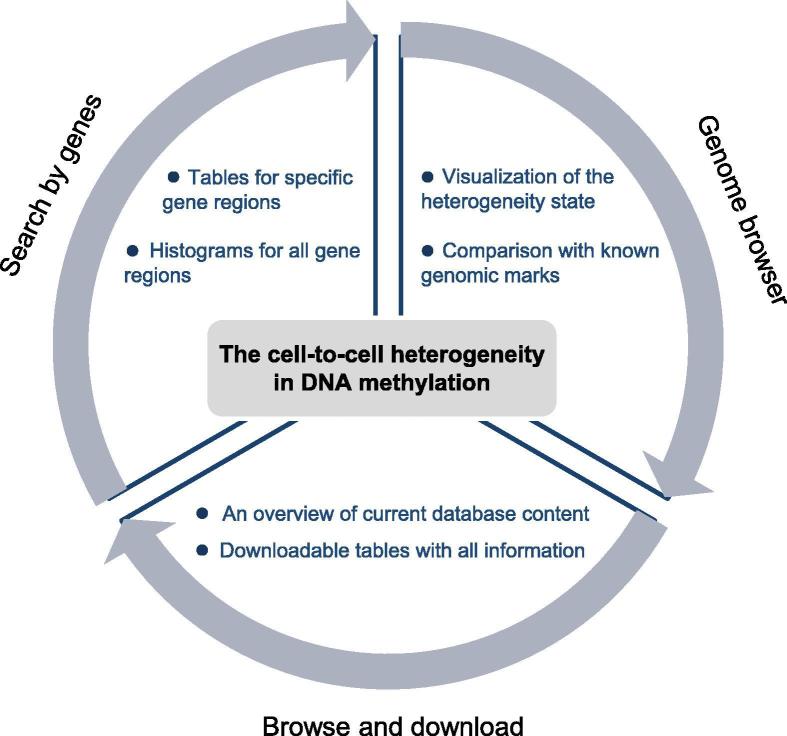
**The functionality of HeteroMeth**

With the computational approach described earlier, we built HeteroMeth, a database of cell-to-cell heterogeneity in DNA methylation calculated from bulk BS-seq data. The functionality of HeteroMeth is shown in Figure 4, including searching, browsing, visualizing, and downloading the data for heterogeneity in DNA methylation for a total of 141 samples in humans, mice, Arabidopsis, and rice.

### HeteroMeth: Search by genes

**Figure 5:**
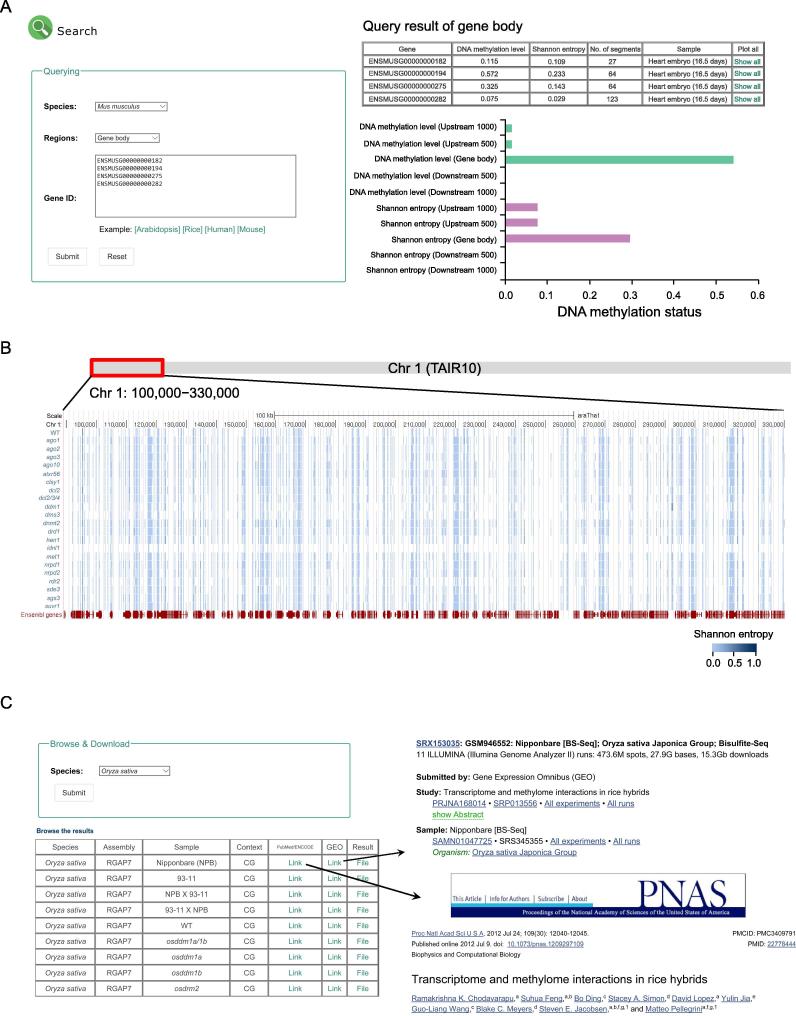
**The interface of HeteroMeth** **A.** After submitting a list of gene IDs, the DNA methylation level and Shannon entropy for each gene can be browsed and downloaded. **B.** The landscapes of heterogeneity in DNA methylation in the wild type Arabidopsis and mutants are shown in the UCSC Genome Browser. A genomic region of Arabidopsis chromosome 1 (Chr 1: 100,000–330,000) is shown. A color gradient from light blue (Shannon entropy = 0) to dark blue (Shannon entropy = 1) shows the magnitude of heterogeneity in DNA methylation. **C.** HeteroMeth provides links to the associated publications or ENCODE experiment ID, and the accession numbers in GEO. Processed HeteroMeth data are also available for downloading.

HeteroMeth allows depicting the heterogeneity in DNA methylation. Data in five regions are provided for each gene annotated in the NCBI Reference Sequence Database (RefSeq), including gene body defined as from the transcription start site (TSS) to the transcription end site (TES), 1000 bp upstream of TSS (Upstream 1000), 500 bp upstream of TSS (Upstream 500), 500 bp downstream of TES (Downstream 500), and 1000 bp downstream of TES (Downstream 1000). After selecting a sample from a species and a region, users can retrieve the DNA methylation information of a group of genes. The output is displayed as a table that contains the DNA methylation level and Shannon entropy of each of these genes (Figure 5A). Three file formats (.csv, .tsv, and .txt) are provided for downloading. In addition, the output page provides a link (by clicking “Show all”) for each gene that displays a histogram of DNA methylation level and Shannon entropy of each of the five aforementioned regions.

**Figure 6:**
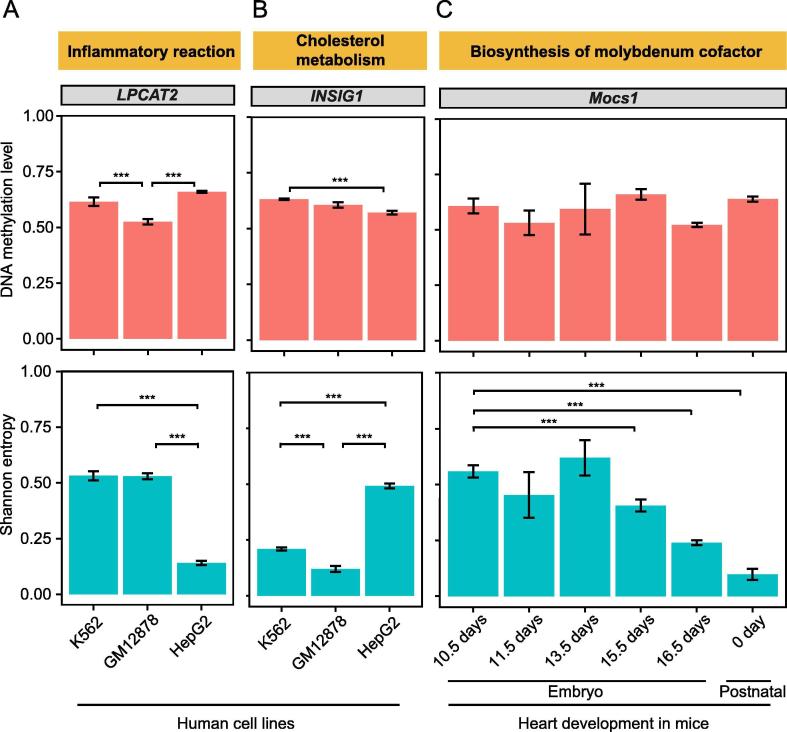
**Examples of genes showing the difference in Shannon entropy between cell lines or developmental stages** **A.***LPCAT2*, a gene related to inflammatory reactions, exhibits similar DNA methylation levels but different Shannon entropy values among three human cell lines including K562, GM12878, and HepG2. **B.***INSIG1*, a gene related to cholesterol metabolism, exhibits similar DNA methylation levels but different Shannon entropy values among three human cell lines including K562, GM12878, and HepG2. **C.***Mocs1*, a mouse gene related to the biosynthesis of molybdenum cofactor, exhibits different Shannon entropy values during the development of mouse heart. Data in embryos of 12.5 and 14.5 days were not shown due to the low quality. Error bars that represent the standard errors of the mean values were calculated with bootstrapping and *P* values were calculated from permutation test. ^***^indicates a significant difference in DNA methylation level or heterogeneity between different samples (*P* < 0.001). LPCAT2, lysophosphatidylcholine acyltransferase 2; INSIG1, insulin induced gene 1; Mocs1, molybdenum cofactor biosynthesis protein 1.

As a proof of concept, we show three genes as examples to illustrate the power of such comparative analysis. We quantified DNA methylation level and Shannon entropy in three human cell lines, K562, GM12878, and HepG2, which were derived from lymphoblast, lymphoblastoid, and liver hepatocellular cells, respectively. *LPCAT2*, a gene related to inflammatory reactions, exhibited significantly higher (*P* < 0.001, permutation test) Shannon entropy in the two immune system-derived cell lines (Figure 6A), whereas *INSIG1*, a gene related to cholesterol metabolism, exhibited significantly higher (*P* < 0.001, permutation test) Shannon entropy in the liver-derived cell line HepG2 (Figure 6B). In contrast, such pattern was not observed for DNA methylation level. More interestingly, *Mocs1*, a gene related to molybdenum cofactor biosynthesis, exhibited reduced heterogeneity (*P* < 0.001, permutation test) in DNA methylation over the developmental progress of the mouse heart, while its DNA methylation level remained largely unchanged (Figure 6C). This may be explained by the role of molybdenum in maintaining the energy mechanism in mitochondria [18]. These observations spur further investigation on this gene for detailed molecular mechanisms. Taken together, these observations suggest that HeteroMeth is a powerful platform to identify unique features of heterogeneity in DNA methylation and to transform BS-seq data into biological knowledge.

### HeteroMeth: Genome browser

For each sample in HeteroMeth, users can browse and visualize the heterogeneity in DNA methylation. After choosing a species, a specific UCSC track hub will provide the heterogeneity in DNA methylation across a genome region for multiple samples (Figure 5B). Users can query the heterogeneity state of a DNA region by entering a gene ID or a genomic location in a chromosome. We use a color gradient from light blue (Shannon entropy = 0, lowest heterogeneity) to dark blue (Shannon entropy = 1, highest heterogeneity) to visualize the magnitude of heterogeneity in DNA methylation (Figure 5B). Users can further access the Shannon entropy of each DNA segment that contains 4 consecutive CpG sites by selecting the “pack” mode. This allows the comparison of DNA methylation heterogeneity with SNPs, CpG islands, other genomic marks, and a variety of third-party annotations.

### HeteroMeth: Browse and download

We also provide a way to browse and download our data. For each species, HeteroMeth shows an overview of the current database content for this species, including the reference genome assembly, the tissues, a short description of the samples, the links to PubMed or the Encyclopedia of DNA Elements (ENCODE), and the accession numbers in the Gene Expression Omnibus (GEO). The heterogeneity state of each DNA segment containing 4 consecutive CpG sites can be downloaded as well (Figure 5C). The tab-delimited file contains the information of the DNA segment, including the chromosome ID, the position in chromosome, DNA methylation level, and Shannon entropy.

## Perspectives

HeteroMeth is the first database for searching, browsing, visualizing, and downloading the heterogeneity in DNA methylation for multiple species, mutants, developmental stages, and tissues. We will update HeteroMeth continuously to incorporate datasets from more species and samples. We will also develop a tool to estimate the heterogeneity of user-provided data. In addition, statistical significance based on permutation test can be incorporated into the database in the future for customized comparisons between the Shannon entropy values of any two DNA segments.

Currently, at least 4 consecutive CpG sites on the same BS-seq read are required for the estimation of the heterogeneity in DNA methylation. Therefore, heterogeneity in DNA methylation can only be assessed in the genomic regions with relative high CpG densities. This hurdle can be overcome when high-throughput sequencing technologies further develop and longer sequencing reads become available. Alternatively, heterogeneity in DNA methylation in lower CpG density regions can be evaluated when the paired-end sequencing reads overlap. HeteroMeth will be updated to include more genomic regions in the future.

BS-seq detects both 5-methylcytosine (5mC) and 5-hydroxymethylcytosine (5hmC) in the genome, whereas the newly developed technology, oxidative BS-seq (oxBS-seq), can detect only 5mC [19]. With more data generated using oxBS-seq, it would be feasible to calculate the heterogeneity in 5mC and 5hmC, respectively. It is of note that the density of 5hmC was much lower than that of 5mC sites [20], and longer sequencing reads are thus required to obtain heterogeneity in 5hmC. We will update HeteroMeth to incorporate this information when available.

## Materials and methods

### Downloading the BS-seq data

Raw reads of scBS-seq data from 20 mESCs cultured in serum were downloaded from GEO under the accession number GSE56879. The corresponding bulk BS-seq data were also downloaded. Raw reads of 141 DNA methylomes were downloaded from GEO [21] and the ENCODE project [22], which include 12 from humans, 26 from mice, 94 from Arabidopsis, and 9 from rice. The genome annotations were retrieved from build GRCh38 [23], build GRCm38, The Arabidopsis Information Resource (TAIR10) [24], and the MSU Rice Genome Annotation Project (RGAP7) [25], respectively. Description of these samples is provided in Table S1.

### Processing of the BS-seq data

The first 9 nucleotides of each read from the scBS-seq data and the corresponding bulk BS-seq data were introduced during the preparation of the high-throughput sequencing library [14]. They, together with the poor-quality bases (Phred score <20) and the adaptor sequence, were trimmed with Trim Galore! (v0.4.4; http://www.bioinformatics.babraham.ac.uk/projects/trim_galore/; --clip_r1 9 --clip_r2 9 --paired). Trimmed reads were further mapped to the reference genome using Bismark (v0.17.0; --bowtie2 --non_directional) [14], [26]. Duplicated reads generated during the library preparation and reads mapped to multiple locations in the genome were discarded afterward.

The bulk BS-seq data in humans, mice, Arabidopsis, and rice were treated similarly. After discarding the poor-quality reads (>50% bases with a Phred score <20) and trimming the adaptor sequences, the trimmed reads (at least 30 bp) were further mapped to their reference genome with Bismark using default parameters.

To estimate the heterogeneity in DNA methylation from bulk BS-seq data, we identified all DNA segments that contain 4 consecutive CpG sites and that were covered by at least 16 sequencing reads. Methylation patterns covered by at least 2 BS-seq reads were identified for each DNA segment and their frequencies were estimated. Methylation patterns with a frequency less than 1/32 likely result from technical errors (*e.g.*, incomplete bisulfite conversion or PCR errors, see main text for details) and were consequently filtered.

### Estimating DNA methylation level and heterogeneity for a DNA region

Shannon entropy and Gini index were calculated to indicate the heterogeneity in DNA methylation as follows [17]:

$$Shannon\;entropy=\frac{1}{b}\sum_{i=1}^{k}\left(-\frac{n_{i}}{N}\log_{2}^{\frac{n_{i}}{N}}\right)$$

where *b* is the number of consecutive CpG sites in a DNA segment (*b* = 4 or 8 in this study); *N* is the number of sequencing reads that cover this segment; *k* is the number of methylation patterns that were observed in this segment; and *n_i_* (*i* ≥ 1 and ≤ *k*) is the number of reads for methylation pattern *i*.

$$Gini\;index=\frac{1}{k-1}\left(k+1-2\left(\frac{\sum_{i=1}^{k}\left(k+1-i\right)n_{i}}{\sum_{i=1}^{k}n_{i}}\right)\right)$$

In the calculation of Gini index for a segment with 4 consecutive CpG sites, we designate *k* as (2^4^ =) 16 because there are a total of 16 possible methylation patterns for 4 consecutive CpG sites. *n_i_* was indexed in non-decreasing order (*n_i_* ≤ *n_i_*_+1_), with *n_i_* designated as 0 if the methylation pattern *i* was not observed in the data.

DNA methylation level was calculated as the fraction of methylated cytosine in total cytosine (^m^C/^m^C + C).

We defined 5 regions for each gene annotated in RefSeq database, including 1000 bp upstream of TSS, 500 bp upstream of TSS, gene body, 500 bp downstream of TES, and 1000 bp downstream of TES. For each region, the DNA methylation level and the Shannon entropy were estimated for all DNA segments containing 4 consecutive CpG sites. The obtained average values were defined as the DNA methylation level and the Shannon entropy of this region, respectively.

### Database implementation

HeteroMeth interfaces to the backend database were organized using PHP and MySQL. HTML5 with JavaScript was used to construct the webpage. The histogram of DNA methylation level and Shannon entropy of all gene regions were created by highcharts.js library (http://www.hcharts.cn/). UCSC Genome Browser [27] was used to visualize the heterogeneity in DNA methylation.

## Authors’ contributions

QH and WQ conceived the study; QH analyzed the data; QH and YZ built the database; QH, SW, and WQ wrote the manuscript. All authors read and approved the final manuscript.

## Competing interests

The authors declare no competing interests.

## Supplementary material

Supplementary Fig. S1 **Permutation test on the heterogeneity in DNA methylation between two DNA segments** Taking the two segments (Chr 2: 98,507,248−98,507,412 and Chr 2: 98,502,437−98,507,595) as shown in Figure 2B for example, we merged all of the epialleles in these two regions and randomly assigned them to these two regions while keeping the number of epialleles unchanged for each segment. We calculated the difference in DNA methylation level (A) and Shannon entropy (B) between these two segments for each permutation (Segment 1−Segment 2). The expected distribution of the difference in DNA methylation level and Shannon entropy were obtained from 1000 permutations and used to calculate *P* values. The observed difference is labelled in red.
