# Supplementary material for: HeteroMeth: A Database of Cell-to-cell Heterogeneity in DNA Methylation
Source: Genomics Proteomics Bioinformatics. 2018 Sep 6;16(4):234–43. doi: 10.1016/j.gpb.2018.07.002 (PMC6203689; doi:10.1016/j.gpb.2018.07.002)
Supplement: Supplementary Fig. S1 — Permutation test on the heterogeneity in DNA methylation between two DNA segments Taking the two segments (Chr 2: 98,507,248−98,507,412 and Chr 2: 98,502,437−98,507,595) as shown in Figure 2B for example, we merged all of the epialleles in these two regions and randomly assigned them to these two regions while keeping the number of epialleles unchanged for each segment. We calculated the difference in DNA methylation level (A) and Shannon entropy (B) between these two segments for each permutation (Segment 1−Segment 2). The expected distribution of the difference in DNA methylation level and Shannon entropy were obtained from 1000 permutations and used to calculate P values. The observed difference is labelled in red. [file mmc1.pdf]

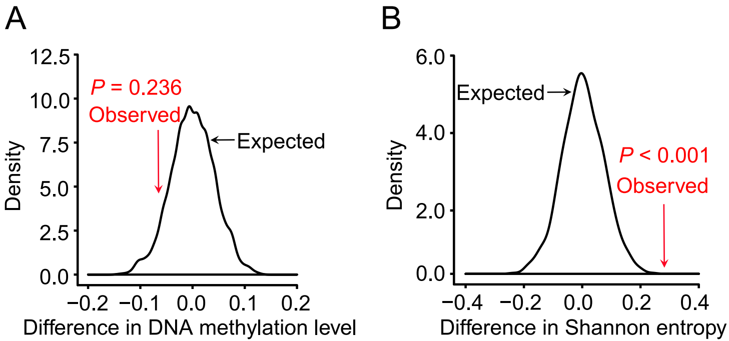

Figure S1. Permutation test on the heterogeneity in DNA methylation between two DNA segments. Taking the two segments (Chr 2: 98,507,248−98,507,412 and Chr 2: 98,502,437−98,507,595) as shown in Figure 2B for example, we merged all of the epialleles in these two regions and randomly assigned them to these two regions while keeping the number of epialleles unchanged for each segment. We calculated the difference in DNA methylation level (**A**) and Shannon entropy (**B**) between these two segments for each permutation (Segment 1 − Segment 2). The expected distribution of the difference in DNA methylation level and Shannon entropy were obtained from 1000 permutations and used to calculate *P* values. The observed difference is labeled in red.
