# Supplementary material for: HeteroMeth: A Database of Cell-to-cell Heterogeneity in DNA Methylation
Source: Genomics Proteomics Bioinformatics. 2018 Sep 6;16(4):234–43. doi: 10.1016/j.gpb.2018.07.002 (PMC6203689; doi:10.1016/j.gpb.2018.07.002)
Supplement: Supplementary Table S1 [file mmc2.docx]

**Table S1 DNA methylomes stored in HeteroMeth**

| Species | Sample ID | Description |
| --- | --- | --- |
| Human | 1001 | Psoas muscle, male adult (34 years old) |
|  | 1002 | Psoas muscle, female adult (30 years old) |
|  | 1003 | Spleen, male adult (34 years old) |
|  | 1004 | Heart left ventricle, male adult (34 years old) |
|  | 1005 | Heart left ventricle, male child (3 years old) |
|  | 1006 | Lung, female adult (30 years old) |
|  | 1007 | Small intestine, female adult (30 years old) |
|  | 1008 | Pancreas, male adult (34 years old) |
|  | 1009 | Pancreas, female adult (30 years old) |
|  | 1010 | HepG2 |
|  | 1011 | K562 |
|  | 1012 | GM12878 |
| Mouse | 2001 | Forebrain, embryo (10.5 days) |
|  | 2002 | Forebrain, embryo (11.5 days) |
|  | 2003 | Forebrain, embryo (12.5 days) |
|  | 2004 | Forebrain, embryo (13.5 days) |
|  | 2005 | Forebrain, embryo (14.5 days) |
|  | 2006 | Forebrain, embryo (15.5 days) |
|  | 2007 | Forebrain, embryo (16.5 days) |
|  | 2008 | Forebrain, postnatal (0 day) |
|  | 2009 | Heart, embryo (10.5 days) |
|  | 2010 | Heart, embryo (11.5 days) |
|  | 2011 | Heart, embryo (12.5 days) |
|  | 2012 | Heart, embryo (13.5 days) |
|  | 2013 | Heart, embryo (14.5 days) |
|  | 2014 | Heart, embryo (15.5 days) |
|  | 2015 | Heart, embryo (16.5 days) |
|  | 2016 | Heart, postnatal (0 day) |
|  | 2017 | Midbrain, embryo (15.5 days) |
|  | 2018 | Hindbrain, embryo (15.5 days) |
|  | 2019 | Liver, embryo (15.5 days) |
|  | 2020 | Embryonic facial prominence, embryo (15.5 days) |
|  | 2021 | Limb, embryo (15.5 days) |
|  | 2022 | Neural tube, embryo (15.5 days) |
|  | 2023 | Intestine, embryo (15.5 days) |
|  | 2024 | Kidney, embryo (15.5 days) |
|  | 2025 | Lung, embryo (15.5 days) |
|  | 2026 | Stomach, embryo (15.5 days) |
| *Arabidopsis* | 3001 | WT |
|  | 3002 | *ago1* mutant |
|  | 3003 | *ago2* mutant |
|  | 3004 | *ago3* mutant |
|  | 3005 | *ago4* mutant |
|  | 3006 | *ago5* mutant |
|  | 3007 | *ago6* mutant |
|  | 3008 | *ago7* mutant |
|  | 3009 | *ago8* mutant |
|  | 3010 | *ago9* mutant |
|  | 3011 | *ago10* mutant |
|  | 3012 | *atxr56* mutant |
|  | 3013 | *bru1* mutant |
|  | 3014 | *clsy1* mutant |
|  | 3015 | *cmt1* mutant |
|  | 3016 | *cmt2* mutant |
|  | 3017 | *cmt3* mutant |
|  | 3018 | *dcl2* mutant |
|  | 3019 | *dcl3* mutant |
|  | 3020 | *dcl4* mutant |
|  | 3021 | *dcl2/4* double mutant |
|  | 3022 | *dcl2/3/4* triple mutant |
|  | 3023 | *ddm1* mutant |
|  | 3024 | *dms3* mutant |
|  | 3025 | *dms4* mutant |
|  | 3026 | *dnmt2 cmt3* double mutant |
|  | 3027 | *dnmt2 drm12* double mutant |
|  | 3028 | *drd1* mutant |
|  | 3029 | *drm1/2* double mutant |
|  | 3030 | *drm1/2 cmt3* triple mutant |
|  | 3031 | *drm3* mutant |
|  | 3032 | *fas2* mutant |
|  | 3033 | *fca* mutant |
|  | 3034 | *fca fpa* double mutant |
|  | 3035 | *fld* mutant |
|  | 3036 | *fpa* mutant |
|  | 3037 | *fve* mutant |
|  | 3038 | *hen1* mutant |
|  | 3039 | *ibm1* mutant |
|  | 3040 | *idn2* mutant |
|  | 3041 | *idn2 idnl1/2* triple mutant |
|  | 3042 | *idnl1 idnl2* double mutant |
|  | 3043 | *ktf1* mutant |
|  | 3044 | *met1* mutant |
|  | 3045 | *met1 cmt3* double mutant |
|  | 3046 | *met1* Het (*met1 ^+/−^* progeny of *met1 ^+/−^* plants) |
|  | 3047 | *met1* WT (*met1 ^+/+^* progeny of *met1 ^+/−^* plants) |
|  | 3048 | *met2* mutant |
|  | 3049 | *mom1* mutant |
|  | 3050 | *msi1* mutant |
|  | 3051 | *nrpb2* mutant |
|  | 3052 | *nrpd1* mutant |
|  | 3053 | *nrpe1* mutant |
|  | 3054 | *rdd* mutant |
|  | 3055 | *rdm1* mutant |
|  | 3056 | *rdr1* mutant |
|  | 3057 | *rdr2* mutant |
|  | 3058 | *rdr6* mutant |
|  | 3059 | *ref6* mutant |
|  | 3060 | *ros3* mutant |
|  | 3061 | *rpa2* mutant |
|  | 3062 | *sde3* mutant |
|  | 3063 | *sde5* mutant |
|  | 3064 | *sdg2* mutant |
|  | 3065 | *sdg8* mutant |
|  | 3066 | *sgs3* mutant |
|  | 3067 | *suvh1* mutant |
|  | 3068 | *suvh2* mutant |
|  | 3069 | *suvh3* mutant |
|  | 3070 | *suvh4/kyp* mutant |
|  | 3071 | *suvh5* mutant |
|  | 3072 | *suvh6* mutant |
|  | 3073 | *suvh4/5/6* triple mutant |
|  | 3074 | *suvh7* mutant |
|  | 3075 | *suvh8* mutant |
|  | 3076 | *suvh9* mutant |
|  | 3077 | *suvh10* mutant |
|  | 3078 | *suvr1* mutant |
|  | 3079 | *suvr2* mutant |
|  | 3080 | *suvr3* mutant |
|  | 3081 | *suvr5* mutant |
|  | 3082 | *suvr1/2/3/4/5* quintuple mutant |
|  | 3083 | *vim1* mutant |
|  | 3084 | *vim2* mutant |
|  | 3085 | *vim3* mutant |
|  | 3086 | *vim1/2/3* triple mutant |
|  | 3087 | +Pi Root 7-day |
|  | 3088 | +Pi Shoot 7-day |
|  | 3089 | −Pi Root 7-day |
|  | 3090 | −Pi Shoot 7-day |
|  | 3091 | +Pi Root 16-day |
|  | 3092 | +Pi Shoot 16-day |
|  | 3093 | −Pi Root 16-day |
|  | 3094 | −Pi Shoot 16-day |
| Rice | 4001 | NPB |
|  | 4002 | 93-11 |
|  | 4003 | NPB × 93-11 |
|  | 4004 | 93-11 × NPB |
|  | 4005 | *osddm1a/1b* mutant (DJ) |
|  | 4006 | WT (DJ) |
|  | 4007 | *osddm1a* mutant (DJ) |
|  | 4008 | *osddm1b* mutant (DJ) |
|  | 4009 | *osdrm2* mutant (DJ) |

*Note*: NPB, Nipponbare; WT, wild type; Het, heterologous; DJ, DongJin.
